# The Microbiota of Recreational Freshwaters and the Implications for Environmental and Public Health

**DOI:** 10.3389/fmicb.2016.01826

**Published:** 2016-11-17

**Authors:** Chang Soo Lee, Minseok Kim, Cheonghoon Lee, Zhongtang Yu, Jiyoung Lee

**Affiliations:** ^1^Division of Environmental Health Sciences, College of Public Health, The Ohio State UniversityColumbus, OH, USA; ^2^Department of Animal Sciences, The Ohio State UniversityColumbus, OH, USA; ^3^Department of Food Science and Technology, The Ohio State UniversityColumbus, OH, USA

**Keywords:** recreational freshwater, bacterial community, environmental factor, public health, pyrosequencing, quantitative PCR

## Abstract

The microbial communities in recreational freshwaters play important roles in both environmental and public health perspectives. In this study, the bacterial community structure and its associations with freshwater environments were investigated by analyzing the summertime microbiomes of three beach waters in Ohio (East Fork, Delaware, and Madison lakes) together with environmental and microbial water quality parameters. From the swimming season of 2009, 21 water samples were collected from the three freshwater beaches. From the samples, 110,000 quality-checked bacterial 16S rRNA gene sequences were obtained and analyzed, resulting in an observation of 4500 bacterial operational taxonomic units (OTUs). The most abundant bacteria were *Mycobacterium* and *Arthrobacter* of the *Actinobacteria* (33.2%), *Exiguobacterium* and *Paenisporosarcina* of the *Firmicutes* (23.4%), *Planktothrix* and *Synechococcus* of the *Cyanobacteria* (20.8%), and *Methylocystis* and *Polynucleobacter* of the *Proteobacteria* (16.3%). Considerable spatial and temporal variations were observed in the bacterial community of *Actinobacteria, Cyanobacteria*, and *Firmicutes*, where the bacterial community structure was greatly influenced by hydrological and weather conditions. The most influential factors were (1) water inflow for *Bacteroidia* and *Clostridia*, (2) turbidity for *Gammaproteobacteria*, (3) precipitation for *Bacilli*, and (4) temperature and pH for *Cyanobacteria*. One noticeable microbial interaction in the bacterial community was a significant negative relationship between *Cyanobacteria* and *Bacilli* (*P* < 0.05). Concerning beach water quality, the level of the genetic markers for cyanobacterial toxin (*mcyA*) was linked to the abundance of *Cyanobacteria*. In addition, unique distributions of the genera *Enterococcus, Staphylococcus, Streptococcus, Bacteroides, Clostridium, Finegoldia, Burkholderia*, and *Klebsiella*, together with a high density of fecal indicator *Escherichia coli*, were markedly observed in the sample from Madison Lake on July 13, suggesting a distinctly different source of bacterial loading into the lake, possibly fecal contamination. In conclusion, deep sequencing-based microbial community analysis can provide detailed profiles of bacterial communities and information on potential public health risks at freshwater beaches.

## Introduction

Bacteria are an important biological component in freshwater ecosystems that play a key role in essential nutrient cycles, such as decomposition of organic compounds (Taylor and Townsend, 2010) and primary production in aquatic food chains (Riemann, 1985). However, some bacteria in recreational freshwaters, such as *Escherichia coli* O157:H7, *Legionella, Pseudomonas*, and *Shigella* have caused public health concerns due to their associations with waterborne illnesses (Hlavsa et al., 2011). Recently, harmful metabolites such as cytotoxic lipopolysaccharides of Gram-negative bacteria and cyanobacterial toxins have increased public health risks (Stewart et al., 2006; Valério et al., 2010).

Many culture-based studies have been conducted to investigate freshwater bacteria, but have provided limited insight into the whole bacterial community due to the limitation that not all bacteria are culturable. Freshwater bacteria have been extensively investigated via culture-independent methods, such as fluorescence *in situ* hybridization (FISH) method (Sekar et al., 2003; Lindström et al., 2005). Terminal restriction fragment length polymorphism (T-RFLP) and quantitative polymerase chain reaction (qPCR) (Eiler and Bertilsson, 2004, 2007; Hu et al., 2016) were used for studying diversity of freshwater bacterial communities associated with cyanobacterial bloom. The application of culture-independent methods provided information on bacterial diversity in freshwaters that had been underestimated in the past. Previous studies revealed that freshwater bacterial community structures have high genetic diversities and high spatiotemporal variation due to their sensitive response to dynamic conditions of aquatic environments. However, some disadvantages still remained due to labor-intensive steps and potential biases in the traditional methods. Recently, many research groups have applied the massive sequencing combined with quantitative analyses, such as qPCR to determine bacterial compositions as well as their abundance in rivers (Staley et al., 2013; Hu et al., 2014; Kolmakova et al., 2014) and lakes (Tseng et al., 2013; Bižić-Ionescu et al., 2014; Winters et al., 2014).

A merit of combined approach with sequencing and quantitative measurement is that the dynamics of specific microbial composition can be examined within the microbial community structure. For example, anthropogenic impact on the water quality of recreational freshwaters can be well understood using various fecal indicating bacteria (Halliday et al., 2014). The influence of nutrients on structural changes of microbial community was well elucidated in freshwater beaches, where changes of bacterial compositions were observed along the nutrient loading gradients depending on sand types (Cloutier et al., 2015). Despite these methodological advances, we still have a limited understanding of how the bacterial community is associated with water quality parameters and environmental conditions in recreational freshwaters, where direct human contact to microbes occur.

Recreational freshwaters provide important public services in many state parks, for example, about 5 million people visited freshwater beaches in the Ohio's state parks in 2009 (Lee et al., 2014). To date, the assessment of public health risk associated with microbial quality has been focused on the use of fecal indicators, such as *E. coli* in freshwater beaches. However, the usage of a single indicator has a limitation in reflecting potential health hazard (Aw and Rose, 2012). The goal of this study was to elucidate whole bacterial community by massive sequencing and to evaluate relationships between the structure of bacterial compositions, water quality and environmental factors during the beach season. For this, the bacterial communities of three recreational freshwaters in Ohio were examined by analyzing pyrosequencing-based bacterial 16S rRNA gene sequences and their associations with the parameters of microbial water quality and environments.

## Materials and methods

### Site, water sampling, and water quality

East Fork Lake (8.7 km^2^; maximum depth 33.4 m) and Delaware Lake (3.9 km^2^; maximum depth 9.1 m) are human-made reservoirs constructed by the United States(US) Army Corps of Engineers for flood control, water supply, recreation, and wildlife management in the Olentangy Watershed. Madison Lake is a small (0.4 km^2^) and shallow (maximum depth 1.8 m) lake for agricultural land use in the Darby Plains of Madison County (Lee et al., 2014). The lakes are often influenced by non-point pollution sources from the surrounding watersheds, including croplands, livestock pasture, forestland, and residential areas. The water quality can be also impacted by human fecal contamination through recreational activities during summer as the lakes are used for recreational purposes, such as swimming beach, picnic areas, and shelterhouses for many visitors (http://ohiodnr.gov).

**Figure 1:**
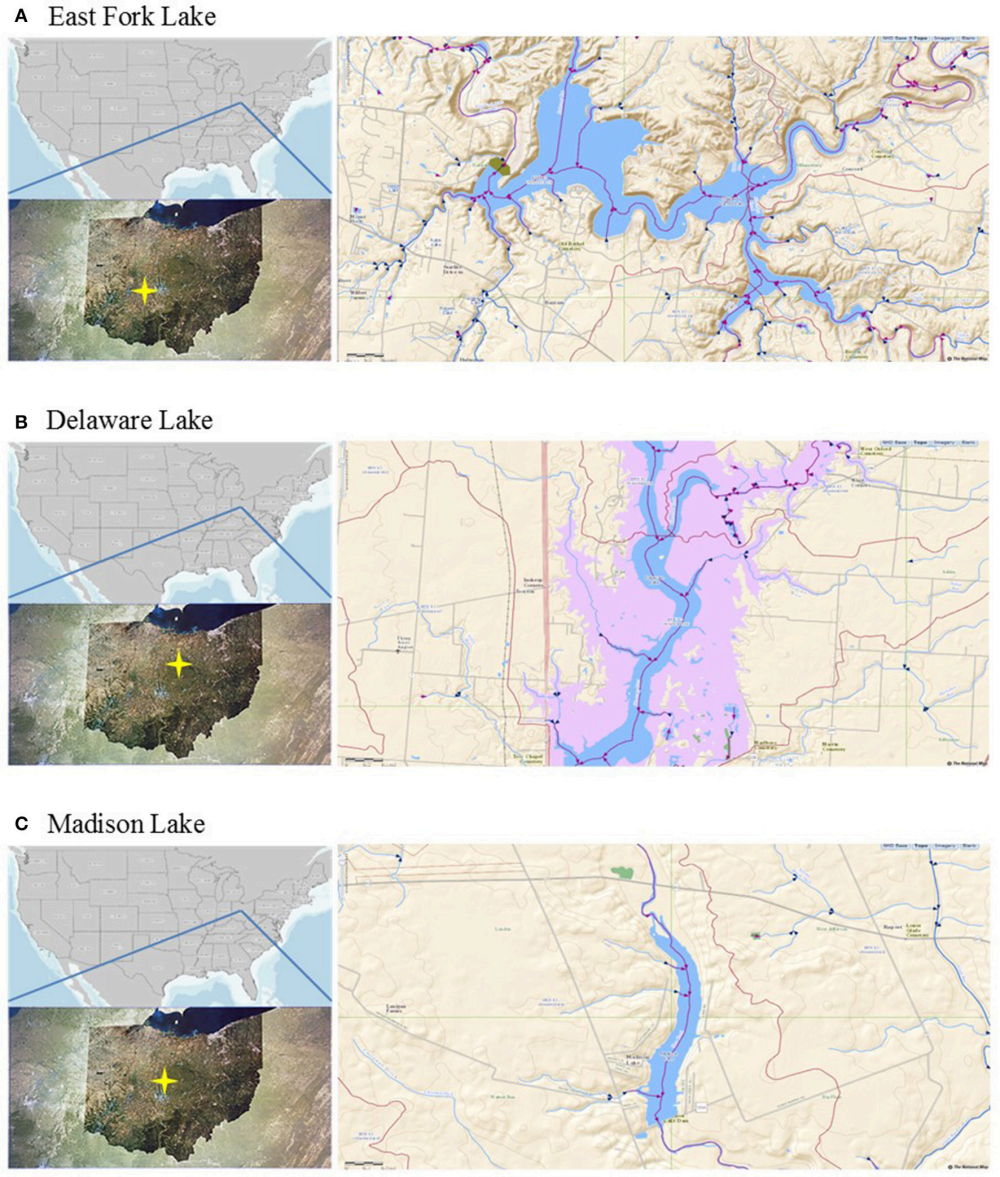
**Locations of the study sites. (A)** East Fork Lake, **(B)** Delaware Lake, and **(C)** Madison Lake in Ohio.

A total of 21 water samples were collected from three recreational freshwaters in Ohio: 9 samples from East Fork Lake (39° 01′ 96.2″ N; 84° 13′ 41.2″ W), 6 samples from Delaware Lake (40° 37′ 14.6″ N; 83° 05′ 87.2″ W), and 5 samples Madison Lake (39° 86′ 97.5″ N; 83° 37′ 43.8″ W; Figure 1). From the surface (~30 cm) near the center of each lake (1-m water depth), a single daily sample (>2 L) was collected in the afternoon (between 12:00 and 18:00) on weekends from June to August 2009 using two autoclaved 1 L bottles (Nalgene, Rochester, NY). Temperature, pH, specific conductivity, and dissolved oxygen level were measured with the YSI 600XL multiprobe data sonde (Yellow Springs Instruments, Yellow Springs, OH) in triplicate. Turbidity was measured with the Hach 2100P portable turbidimeter [(US Environmental Protection Agency (USEPA), 1983)]. The sampling was carried in accordance with the recommendations of the Ohio Department of Health Sampling Guideline.

After transportation to the laboratory on ice, a 200 mL aliquot of each water sample was filtered through a 47-mm-diameter, 0.22-μm-pore-sized mixed cellulose ester membrane filter (Millipore, Bedford, MA) to collect bacteria (Yannarell and Triplett, 2005). The membrane filter was transferred into a 50 mL sterile tube and then stored at −80°C until further analysis. To fecal indicator levels, *E. coli* densities were determined in triplicate within 6 h after transportation to the laboratory according to USEPA Method 1603 (USEPA, 2002). Precipitation data were obtained local weather stations of the National Weather Service (https://www.ncdc.noaa.gov/data-access). Water inflow data were obtained from the Louisville District of the US Army Corps of Engineers (http://www.lrl.usace.army.mil/) and the National Water Information System of the US Geological Survey (http://waterdata.usgs.gov/oh/nwis/rt).

### DNA extraction

Bacterial DNA was extracted from the cellulose membrane as described previously (Wolcott et al., 2009). Briefly, the membrane filter was cut into two pieces using sterile scalpel blades and each piece was transferred into a sterile microcentrifuge tube containing 500 μL RLT buffer with β-mercaptoethanol (Qiagen, Valencia, CA). For complete bacterial lysis, a bead-beating method was performed using a Qiagen TissueLyser at 30 Hz for 5 min with a sterile 5 mm steel bead (Qiagen) and 500 μL sterile 0.1 mm glass beads (Scientific Industries, Inc., NY). After centrifugation at 14,000 rpm for 30 s, 100 μL supernatant aliquots from each tube were transferred into a new sterile microcentrifuge tube. Thus a total of 200 μL supernatatnts were prepared in the same tube and subjected to DNA extraction using a Qiagen Stool Kit (Qiagen). Extracted DNA was eluted from the DNA spin column into 30 μL DNA/RNA-free water and diluted to a final concentration of 20 ng/μL using a NanoDrop spectrophotometer (Nyxor Biotech, Paris, France). Positive control for the DNA extraction was performed by spiking *Bacteroides fragilis* ATCC 25285^T^ into 200 ml of sterile phosphate buffered saline (PBS; 0.14 M NaCl, 2.7 mM KCl, 10.1 mM Na_2_HPO_4_, 1.8 mM KH_2_PO_4_ [pH 7.6]) as described previously (Lee et al., 2014). For a negative control, 200 mL of sterile phosphate buffered saline without spiking was filtered through a membrane filter and the DNA extraction was carried out from the membrane filter, resulting in no false-positive signals.

### Pyrosequencing and sequencing data pipeline

Bacterial tag-encoded pyrosequencing was performed at the Research and Testing Laboratory (Lubbock, TX) using the Roche 454/FLX system according to the manufacturer's instructions (Roche, Indianapolis, IN). The 16S universal eubacterial primers Gray28F (5′-GAGTTTGATCNTGGCTCAG-3′) and Gray519R (5′-GTNTTACNGCGGCKGCTG-3′) were used to amplify up to the 520 bp V1-V3 region of the 16S rRNA gene as described previously (Dowd et al., 2008). The resultant amplicons were analyzed through a pipeline of the Research and Testing Laboratory (http://www.researchandtesting.com/docs/Data_Analysis_Methodology.pdf). Briefly, noisy sequence-reads were initially removed by screening for low quality (<25 Phred scores), short length (<250 bps), and long homopolymers (>6 bp). Singleton sequences and ambiguous bases in the regions of the primer and barcode were removed. Prefix de-replication, clustering, and centroid sequence generation were performed using the USEARCH algorithm (Edgar, 2010). Further non-flow based denoising was performed using the Acacia software (ver. 1.52) (Bragg et al., 2012) to correct errors. Analysis of the sequencing data was performed using bioinformatics programs, as implemented in the QIIME software package (ver. 1.4.0) (Caporaso et al., 2010b). Sequences were aligned against the Greengenes core set database using the PyNAST option (Caporaso et al., 2010a), and chimeric sequences were removed from the aligned sequences using the ChimeraSlayer option (Haas et al., 2011). Quality-checked sequences were taxonomically assigned based on the Ribosomal Database Project (RDP) naïve Bayesian rRNA Classifier v2.2 (Wang et al., 2007) and clustered into operational taxonomic units (OTUs) at 97% sequence similarity as a cut-off value for the species assignment using the UCLUST option (Edgar, 2010). For a more detailed taxonomical classification, the sequences assigned to 13 tentative *Cyanobacteria* genera (GpI to GPXIII) were reclassified based on the National Center for Biotechnology Information (NCBI) database. All pyrosequencing reads obtained in this study were submitted to the NCBI Sequence Read Archive (SRA) under the study accession number SAMN02087719–SAMN02087739.

### Diversity estimates and statistical analysis

The observed OTUs, estimated OTUs (Chao1 and ACE), and alpha diversity indices (Shannon and Simpson) were calculated with normalized sequence reads using the QIIME software (Caporaso et al., 2010b). The maximum number of OTUs and coverage percentages were calculated from rarefaction curves using the non-linear model procedure (PROC NLIN) of the SAS software (V9.1; SAS Institute, Cary, NC) as described previously (Kim et al., 2011). Principal coordinates analysis (PCoA) was conducted using the weighted UniFrac distance matrix of a phylogenetic tree constructed with representative OTUs (Lozupone and Knight, 2005), which was compared with the hierarchic clustering constructed by the hclust function in the R software package using average linkage algorithms (UPGMA) (R Development Core Team, 2009).

To assess potential associations among environmental, fecal indicator, and pyrosequencing data sets, Spearman correlation and canonical correlation analyses (CCA) were conducted using the R package (ver. 2.15.1) (González et al., 2008). The data for the microbiological terms, including fecal indicator levels and pyrosequencing reads, were log (base 10)-transformed. To compare non-normally distributed data of environmental parameters among the three lakes, the Kruskal-Wallis test was performed using the R package, where significant differences and tendencies were set to *P* < 0.05 and 0.1, respectively.

### Quantitative PCR (qPCR)

For the evaluation of toxic cyanobacteria, one of microcystin biosynthesis gene cluster, *mcy*A gene, was measured in triplicate by a SYBR qPCR assay using a primer set (forward M1rF: 5′-AGCGGTAGTCATTGCATCGG-3′, reverse M1rR: 5′-GCCCTTTTTCTGAAGTCGCC-3′) (Yoshida et al., 2007). For the quantification of the *mcyA* genetic marker, the DNA of a *Microcystis aeruginosa* NIES-843 culture (as a positive control; kindly provided by Dr. Wilhelm, University of Tennessee) was extracted by the xanthogenate nucleic acid isolation method (Tillett and Neilan, 2000). The *mcyA* qPCR assay was carried out in a total volume of 20 μL qPCR mixture containing a 2μL of *M. aeruginosa* DNA template, 10 μL of SYBR universal PCR master mix (Applied Biosystems, Foster City, CA), and a 200 nM primer set. Thermal cycling was conducted for an initial 95°C for 10 min, followed by 40 cycles of denaturation at 95°C for 30 s, and annealing and extension at 62°C for 3 min using the ABI 48-well StepOne™ Real Time System (Applied Biosystems, Foster City, CA).

To check specificity of qPCR amplicons in the assay, a melting point analysis was carried out by lowering the temperature to 50°C, and then raising it by 1.0°C/s until it reached 95°C. A mixture of all PCR reagents without DNA/RNA templates was used as a negative control, resulting in no false-positive signals. For the standard curve, the PCR products of *mcyA* gene were cloned using the pGEM-T Vector System (Promega Co., Madison, WI), followed by the blue-white screening and sub-culturing according to the manufacturer's instruction. The plasmid DNAs of sub-cultured clones were extracted using QIAprep Spin Miniprep Kit (Qiagen, Valencia, CA). The concentration of DNA was measured with NanoDrop spectrophotometer and each 10-fold serial dilution was then prepared using linearized plasmid DNA, followed by each qPCR assay to construct each standard curve with plotting C_T_ values vs. log_10_ values of the gene copy number, where a limit of quantification (LOQ) was defined as the lowest gene copy number within the linear range of quantification (Lee et al., 2015).

## Results

### Environmental and microbial water qualities

Environmental parameters of water samples are summarized by lake (Table 1). In general, dissolved oxygen, turbidity, water inflow, and precipitation had normal variations without significant differences among the three lakes. However, higher pH and lower specific conductivity were found in East Fork Lake compared to the other two lakes (*P* < 0.001). The temperature of East Fork Lake tended to be higher than those of the two other lakes (*P* < 0.1).

**Table 1:** **Environmental and microbial water qualities in three recreational freshwaters**.

**Site**	**East Fork Lake**	**Delaware Lake**	**Madison Lake**
**Sample number**	***n* = 9**	***n* = 5**	***n* = 7**
Location	39° 01′ 96.2″ N	40° 37′ 14.6″ N	39° 86′ 97.5″ N
	84° 13′ 41.2″ W	83° 05′ 87.2″ W	83° 37′ 43.8″ W
Temp (°C)	27.58 ± 2.22	25.22 ± 1.62	25.97 ± 1.50
Ph	9.14 ± 0.17	8.73 ± 0.07	8.81 ± 0.18
Specific Conductivity (μS/cm)	270 ± 4	486 ± 3	482 ± 45
Dissolved oxygen (mg/L)	9.19 ± 2.83	10.08 ± 2.17	10.52 ± 2.75
Turbidity (NTU)	35.1 ± 37.0	14.3 ± 4.4	38.3 ± 18.2
Water flow (m^3^/s)	5.30 ± 6.86	1.12 ± 1.19	2.63 ± 3.00
Precipitation (mm)	1.83 ± 5.12	3.40 ± 7.05	0.04 ± 0.10
*E. coli* (CFU/100 mL)	30.6 ± 8.4	29.3 ± 1.6	52.7 ± 6.4
*mcyA* (gene copies/100 mL)	7.8 ± 21.5	9.2 ± 15.4	2.3 ± 7.5

For microbial water quality parameters, higher geometric means of *E. coli* were found in Madison Lake compared to East Fork Lake or Delaware Lake (*P* < 0.05). *E. coli* densities of 18 out of the 21 samples remained below the single-sample advisory limit in freshwater beaches (235 CFU/100 mL) defined by USEPA advisory. Three high *E. coli* densities above the advisory limit were observed from two East Fork Lake samples (Aug 1 and 2) and one Madison Lake sample on July 13 (Supplementary Figure 1A). Levels of *mcyA* genetic marker ranged from undetected to 4.3 × 10^3^ gene copies/100 mL; positive detections were observed from two East Fork Lake samples (June 21 and June 27), two Delaware Lake samples (July 20 and July 21), and one Madison Lake sample (June 24) (Supplementary Figure 1B).

The level of *mcyA* genetic marker was calculated using a standard curve. The standard curve showed a good linear range of quantification from 4.4 × 10^0^ to 4.4 × 10^8^ gene copies/reaction (*R*^2^ = 0.9963). The slope, y-intercept, and amplification efficiency of the standard curve were −3.5334, 35.728, and 91.8%, respectively. The limit of quantification was determined as 4.4 × 10^0^ gene copies/reaction (Supplementary Figure 2).

### Bacterial diversity and richness

A total of 108,566 quality-checked sequences (average length 430 bp; average Phred score 34) were retrieved from 21 total samples from three lakes. To normalize read number variation among samples, raw sequences in the range of 3396–7141 per sample were normalized into the minimum number (3396) by selecting proportional percentages from each dataset with the subsample function in the QIIME. The re-sampled dataset was used to estimate the bacterial diversity and abundance. At the species level, defined by 97% sequence similarity, the numbers of OTUs ranged from ~300 to 700; where the coverage ratio of observed OTUs to estimated OTUs ranged from 66 to 88%. The Shannon and Simpson indices of samples ranged from 2.641 to 4.564 and 0.053 to 0.228 at 97% sequence similarity, respectively (Table 2).

**Table 2:** **Bacterial diversity index by analyzing 16S rRNA gene sequences based on a cut-off <97% sequence identity for the delineation of operational taxonomic units (OTUs) in three recreational freshwaters**.

**Lake**	**Date**	**Raw read**	**Covered OTUs (%)**	**Estimated OTUs**			**α-Diversity index**	
				**Non-linear**	**Chao1**	**ACE**	**Shannon**	**Simpson**
East Fork	06/21/09	6152	471 (78)	605	1035	1477	3.578	0.108
	06/27/09	4803	606 (73)	831	1231	1991	4.069	0.071
	07/26/09	7141	571 (80)	714	1100	1446	3.352	0.177
	08/01/09	7068	662 (80)	823	1172	1716	3.931	0.099
	08/02/09	4734	697 (76)	912	1464	2016	4.564	0.053
	08/08/09	4085	599 (78)	770	1211	1869	4.322	0.075
	08/15/09	4126	463 (76)	608	912	1383	3.377	0.205
	08/16/09	5522	562 (82)	689	1026	1012	3.608	0.179
	08/22/09	4619	548 (77)	711	1186	1707	4.047	0.067
Delaware	06/11/09	7127	303 (88)	343	482	579	2.848	0.185
	06/16/09	5184	401 (75)	538	816	1305	2.974	0.218
	07/13/09	3396	459 (66)	691	1089	1896	3.909	0.067
	07/20/09	4051	468 (70)	671	925	1650	3.658	0.085
	07/21/09	5202	554 (67)	824	1367	2349	3.638	0.089
Madison	06/24/09	5037	567 (77)	741	1049	1354	3.351	0.163
	06/30/09	4166	348 (86)	403	593	734	3.833	0.066
	07/06/09	5363	306 (87)	353	540	610	2.641	0.228
	07/13/09	5102	483 (74)	656	1317	1838	3.235	0.186
	07/14/09	4732	570 (77)	743	1089	1538	4.132	0.070
	07/21/09	5834	534 (77)	696	1007	1566	3.761	0.078
	07/27/09	5122	527 (75)	707	1039	1683	3.923	0.066

**Figure 2:**
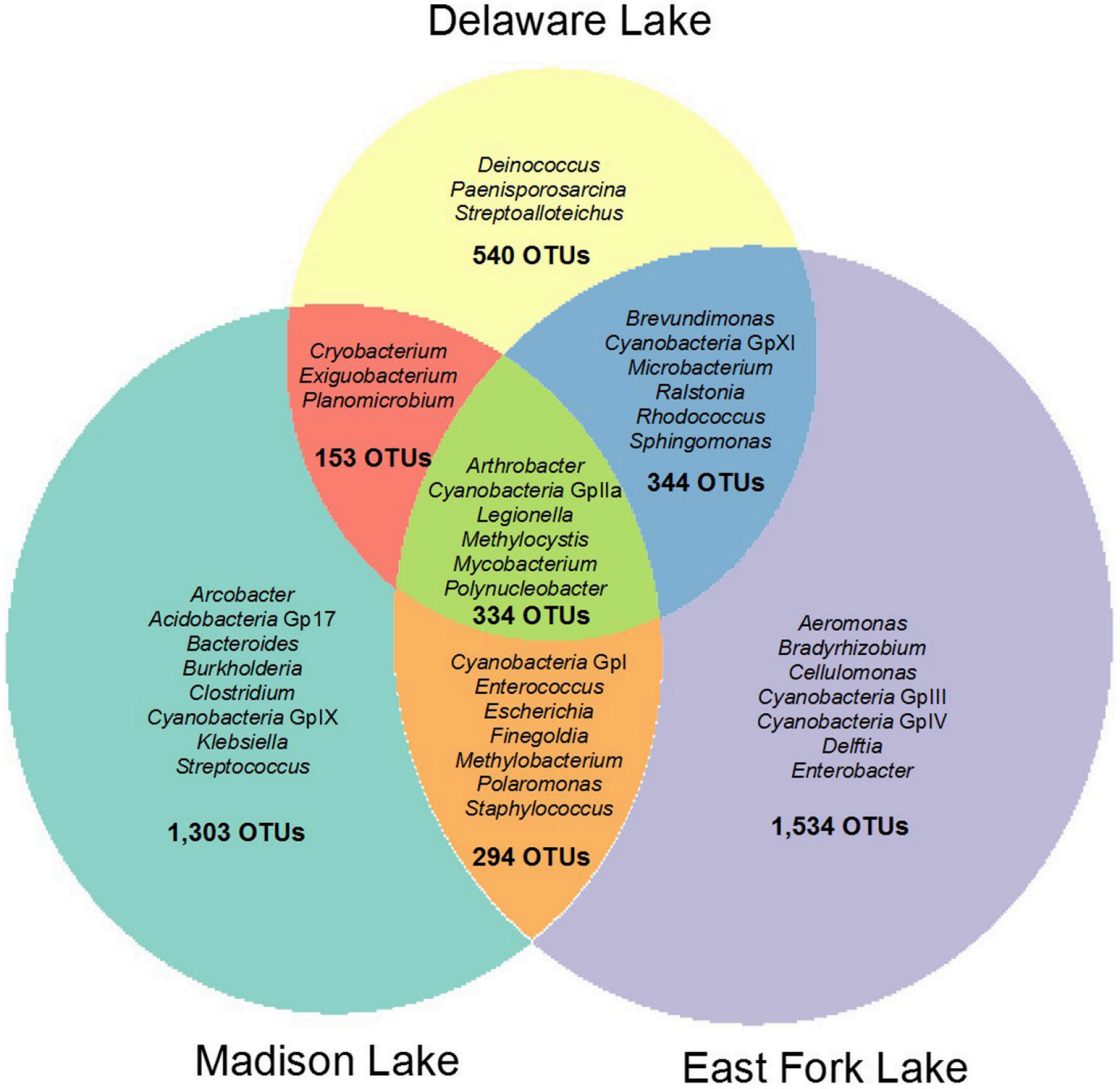
**Weighted Venn diagram representing shared and unique bacterial compositions in three recreational freshwaters**. The bacterial OTUs at the genus levels were defined with 16S rRNA gene sequences based on a cut-off value of >97% sequence similarity.

Total, shared, and unique OTUs from the three lakes are illustrated in a weighted Venn diagram (Figure 2). East Fork Lake had the most unique bacteria (1534 OTUs), followed by Madison Lake (1303 OTUs) and Delaware Lake (540 OTUs). Shared bacterial components were observed; 678 OTUs between East Fork Lake and Delaware Lake, 628 OTUs were shared between East Fork Lake and Madison Lake, and 487 OTUs between Delaware Lake and Madison Lake, respectively. Common bacteria found in all three lakes were 334 OTUs, which belonged to the genera *Cyanobacteria* GpIIa, *Legionella, Methylocystis, Mycobacterium*, and *Polynucleobacter* (Figure 2).

### Distribution of taxa and phylotypes

**Figure 3:**
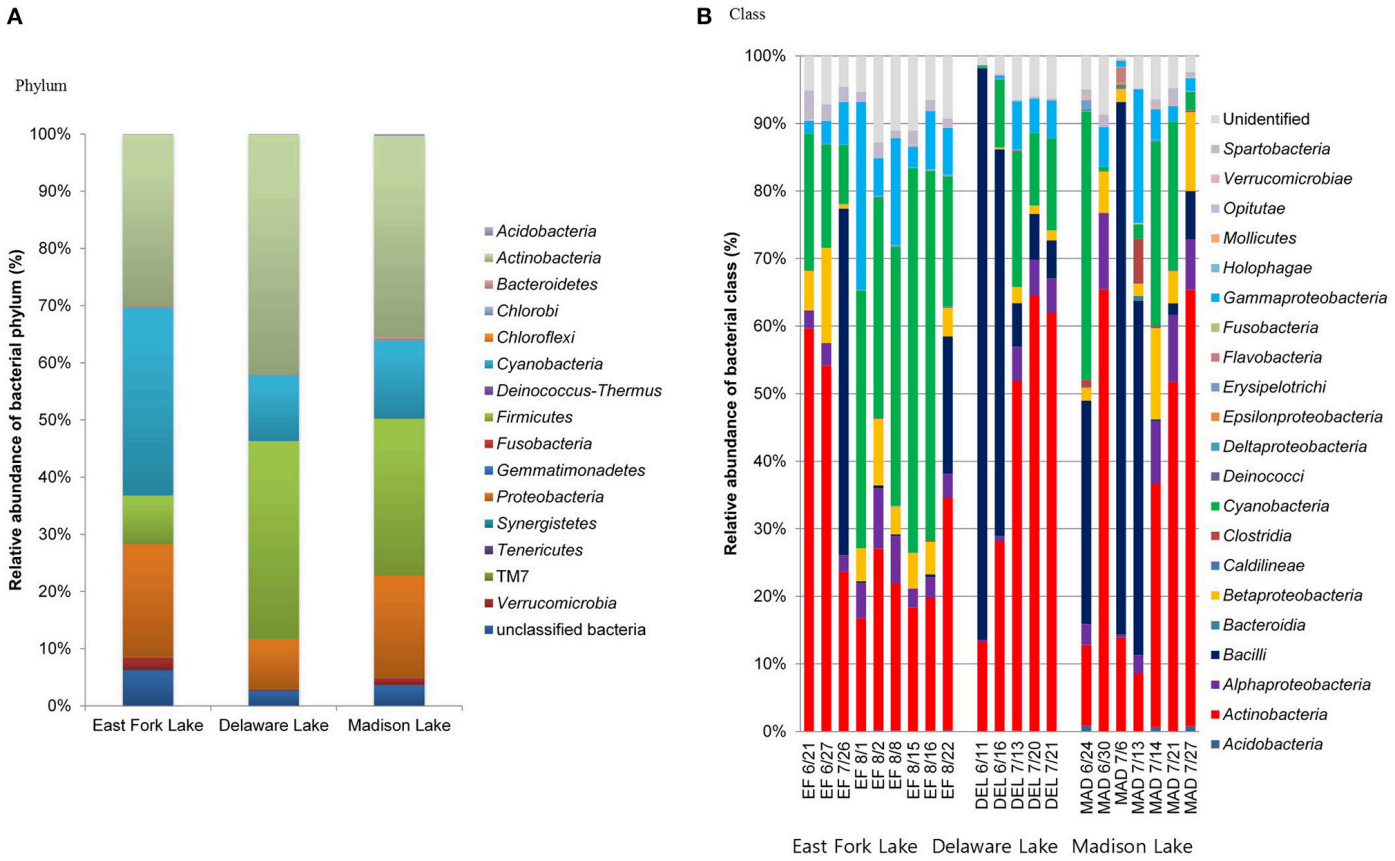
**Relative abundances of phylogenetic groups at (A)** phylum and **(B)** class levels found in water samples from the three lakes.

The microbial communities included four dominant phyla (*Actinobacteria, Cyanobacteria, Firmicutes*, and *Proteobacteria*) with more than 95% of the total population, as well as less abundant phyla (*Acidobacteria, Bacteroidetes, Chlorobi, Chloroflexi, Deinococcus*-*Thermus, Fusobacteria, Gemmatimonadetes, Planctomycetes, Synergistetes, Tenericutes, Verrucomicrobia*, and a candidate phylum TM7) with <5% of the total population. In general, *Actinobacteria* were most abundant in the three lakes (30.0% in East Fork Lake, 41.9% in Delaware Lake, and 35.2% in Madison Lake. *Cyanobacteria* and *Firmicutes* were also dominant; however, their abundances differed between East Fork Lake and the other two lakes. *Cyanobacteria* were more abundant in East Fork Lake (33.0%) than in the other lakes (11.6% in Delaware Lake and 13.8% in Madison Lake), while *Firmicutes* were less abundant in East Fork Lake (8.5%) than the other lakes (34.7% in Delaware Lake and 27.3% in Madison Lake). *Proteobacteria* accounted for 19.8% in East Fork Lake, 8.7% in Delaware Lake, and 17.9% in Madison Lake (Figure 3A). Temporal variations were observed in the relative abundances of some bacterial classes such as *Actinobacteria, Bacilli*, and *Cyanobacteria*; the class *Bacilli* had the most variation, ranging from 0.1 to 84.6% of the total population. On the other hand, *Alpha*-, *Bet*a-, and *Gammaproteobacteria* showed less fluctuation in their abundances across all water samples, irrespective of lake, compared to the other dominant classes (Figure 3B).

**Figure 4:**
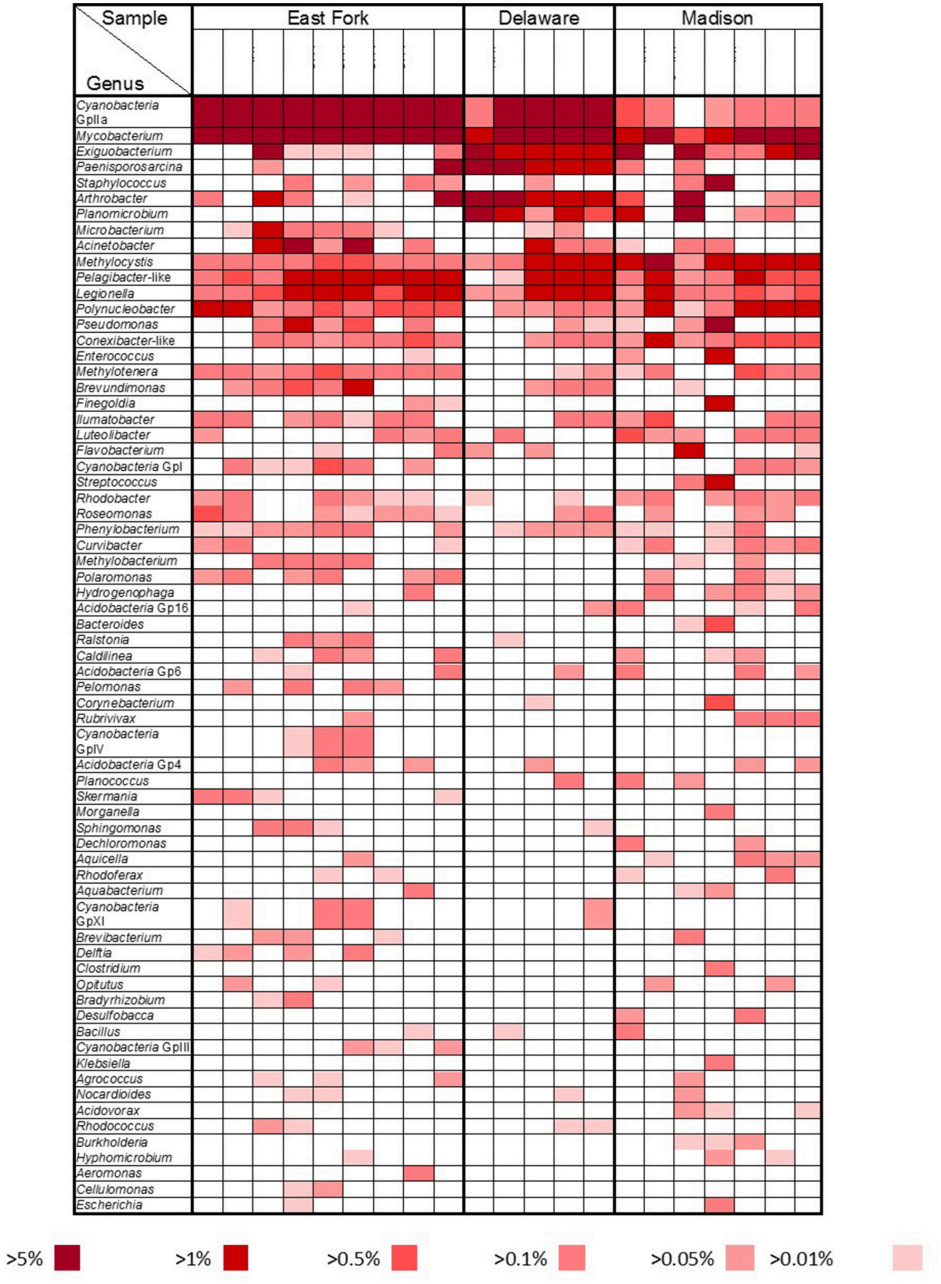
**Bacterial community heat map**. Relative abundances at the genus level were compared by color gradients among three recreational freshwaters (East Fork Lake, Delaware Lake, and Madison Lake). Percentages below the table indicate the abundance of each genus relative to total bacteria.

A more detailed bacterial community structure is illustrated by a heat-map at the genus level (Figure 4). Briefly, dominant members (accounting for >5% of the total sequences) were: *Cyanobacteria* GpIIa (29.1%), *Mycobacterium* (13.4%), and *Exiguobacterium* (7.5%) in East Fork Lake; *Paenisporosarcina* (27.7%), *Mycobacterium* (18.5%), *Cyanobacteria* GpIIa (9.1%), *Exiguobacterium* (7.2%), and *Arthrobacter* (5.7%) in Delaware Lake; and *Mycobacterium* (12.6%), *Exiguobacterium* (12.5%), and *Staphylococcus* (6.9%) in Madison Lake.

#### Actinobacteria

*Mycobacterium* was the most dominant genus of *Actinobacteria*. The most representative OTUs, #237, #4161, and #1502, were identified as *Mycobacterium moriokaense, Mycobacterium wolinskyi*, and *Mycobacterium brisbanense*, respectively (Supplementary Table 1). In addition, OTU #1023, identified as *Mycobacterium arupense*, and OTU #1782, identified as *Mycobacterium senegalense*, were more frequently found in East Folk Lake than in the other two lakes.

The second most abundant genus of *Actinobacteria, Arthrobacter*, accounted for 4.0% of the *Actinobacteria*. The genus *Arthrobacter* was represented as OTU #2064, which was most closely related to *Arthrobacter oryzae*. The genus *Arthrobacter* was frequently found in Delaware Lake (3.2% of the total sequences), but less frequently in East Fork Lake and Madison Lake (0.1% of the total sequences; Supplementary Table 1).

#### Cyanobacteria

*Cyanobacteria* GpIIa was the most abundant genus of *Cyanobacteria* (55.0%). Reclassification of *Cyanobacteria* GpIIa indicated that the most representative OTUs were *Synechococcus rubescens* and *Planktothrix rubescens* (Supplementary Table 1). *Synechococcus* was a dominant component in East Fork Lake (87.9%) and Delaware Lake (73.5%), while it was rare in Madison Lake (1.0%). The genus *Cyanobium* accounted for 8.8 and 20.1% of *Cyanobacteria* in East Fork Lake and Delaware Lake, respectively, but only 0.7% in Madison Lake. On the other hand, the genus *Planktothrix* was a dominant component of *Cyanobacteria* in Madison Lake (93.8%), but was a rare component in East Fork Lake (0.6%), and absent in Delaware Lake.

#### Firmicutes

*Exiguobacterium* and *Paenisporosarcina* were the most dominant *Firmicutes* genera among three lakes, accounting for 40.6 and 35.0% of *Firmicutes*, respectively. In addition, the genera *Staphylococcus* and *Planomicrobium* accounted for ~10% of *Firmicutes*. *Exiguobacterium* is one of the most abundant *Firmicutes* genera (20.9%); OTUs #3900 and #4281 were most closely related to *Exiguobacterium acetylicum* and *Exiguobacterium undae* DSM 14481^T^, respectively. Other dominant *Exiguobacterium* were OTUs #249 and #4145, which were identified as *Exiguobacterium mexicanum* and *Exiguobacterium artemiae*, respectively.

*Paenisporosarcina* accounted for 117 of the 502 *Firmicutes* OTUs; the representative OTU #4340 was identified as *Paenisporosarcina macmurdoensis*. Another four dominant *Paenisporosarcina* found mainly in Delaware Lake were OTUs #2029, #2965, #1195, and #1305, which were all closely related to *P. macmurdoensis* (Supplementary Table 1). *Enterococcus* and *Staphylococcus* were generally rare; they were primarily found in one sample from Madison Lake on July 13, where their representative OTUs were closely related to *Enterococcus faecalis* and *Staphylococcus aureus* subsp. *aureus*, respectively (Supplementary Table 1).

#### Proteobacteria

*Acinetobacter* was the most dominant genus of *Proteobacteria*, accounting for 11.9% of all the *Proteobacteria* sequences. *Acinetobacter* were mainly found in East Fork Lake. *Methylocystis, Pelagibacter*-like, *Pseudomonas, Legionella*, and *Methylotenera* were frequently observed in all three lakes, accounting for ~8.9, 7.6, 5.0, 3.2, and 1.9% of *Proteobacteria*, respectively. *Polaromonas* were found in East Fork Lake and Madison Lake, accounting for 1.2% of *Proteobacteria*. The remaining known genera each accounted for <1.0% of *Proteobacteria*, but approximately half of the *Proteobacteria* sequences could not be assigned to any known genus. The most abundant unclassified group belonged to the family *Legionellaceae*, accounting for 11.5% of *Proteobacteria*.

Representative OTUs for *Methylocystis* and *Pelagibacter*-like were OTU #3420, identified as *Methylocystis heyeri*, and OTU #1182, most closely related to *Pelagibacter ubique*. A representative OTU for *Polynucleobacter* was OTU #464, identified as *Polynucleobacter acidiphobus*. Two abundant *Pseudomonas* were OTUs #2815 and #2611, identified as *Pseudomonas aeruginosa* and *Pseudomonas cedrina* subsp. *fulgida*, respectively (Supplementary Table 1).

### Statistical analysis of the bacterial community structure

In the Spearman correlation analysis of pyrosequencing reads, a significant positive correlation was found at the genus level between *Cyanobacteria* GpIIa and temperature in all lakes (*r* = 0.514, *P* = 0.017). Within bacterial compositions at the class level, *Cyanobacteria* and *Bacilli* had a negative relationship with each other (*r* = −0.526, *P* = 0.014). In addition to the negative relationship between *Cyanobacteria* GpIIa and *Methylocystis* in all lakes (*r* = −0.454, *P* = 0.039), *Cyanobacteria* GpIIa was negatively related to *Arthrobacter* (*r* = −0.590, *P* = 0.026) in Madison Lake and with *Exiguobacterium* (*r* = −0.895, *P* = 0.040) or *Planomicrobium* (*r* = −0.977, *P* = 0.004) in Delaware Lake (Table 3).

**Table 3:** **Significant correlations (A) between temperature and bacterial OTUs at the genus level and (B) within bacterial compositions**.

**(A)**		
**Bacteria (genus level)**	**Temperature**	
	**Correlation coefficient (*r*)**	***P*-value**
*Cyanobacteria* GpIIa	0.514	0.017
*Roseomonas*	0.487	0.025
*Peredibacter*	0.437	0.048
*Paenisporosarcina*	−0.485	0.026
*Arthrobacter*	−0.547	0.010
**(B)**		
**Bacteria (genus level)**	***Cyanobacteria* GpIIa**	
	**Correlation coefficient (*r*)**	***P*-value**
*Methylocystis*	−0.454^A^	0.039
*Arthrobacter*	−0.590^M^	0.026
*Exiguobacterium*	−0.895^D^	0.040
*Planomicrobium*	−0.977^D^	0.004

*A, all lake; M, Madison Lake; D, Delaware Lake*.

**Figure 5:**
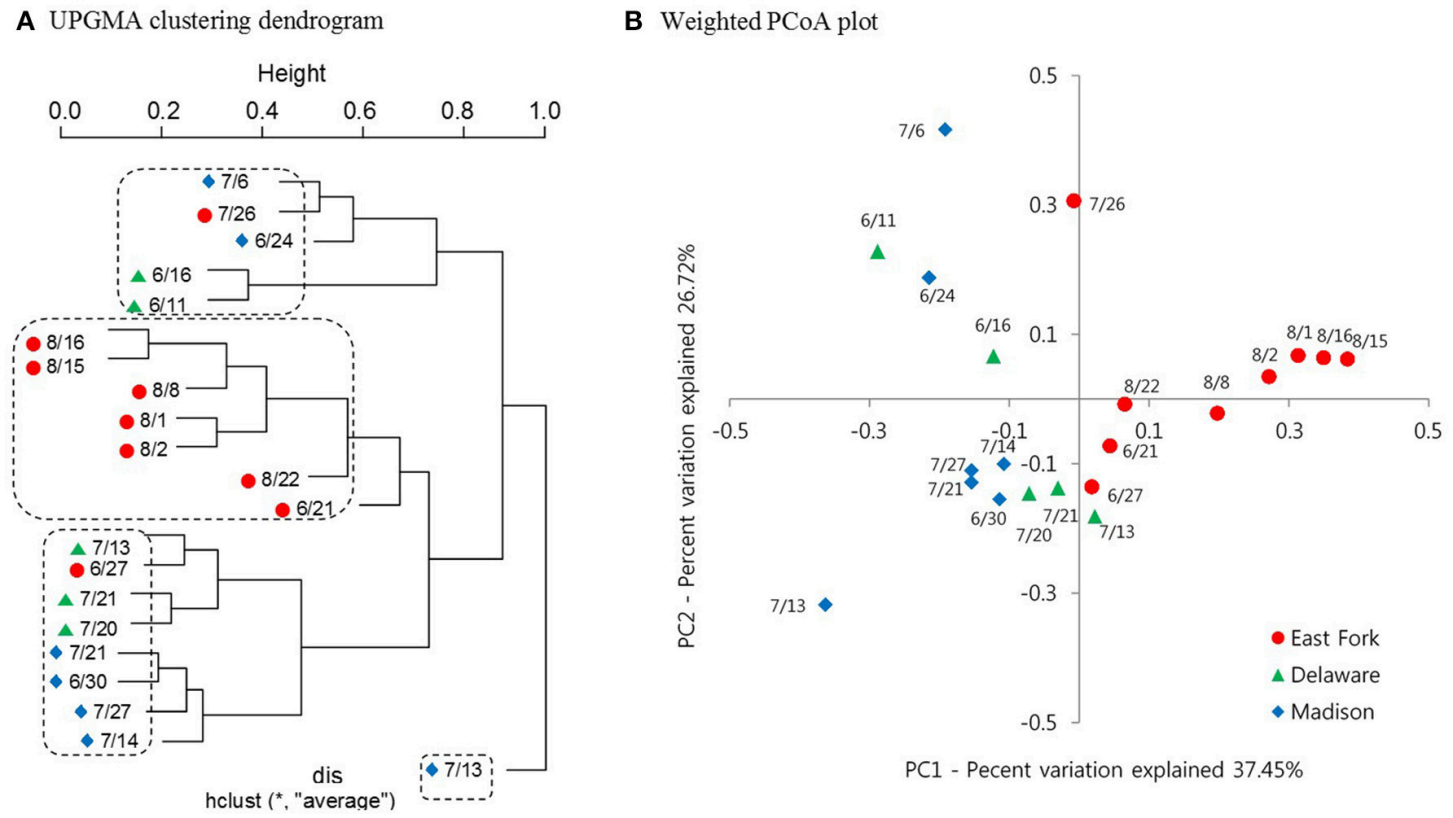
**Clustering dendrogram and Weighted PCoA plot representing the bacterial community structure of three recreational freshwaters. (A)** Average linkage (UPGMA) clustering dendrogram showing similarities among 21 pyrosequencing datasets from 21 samples. **(B)** Weighted PCoA plot representing UniFrac distance matrix of phylogenetic trees constructed from represented OTUs. Samples from three lakes are indicated by different colors: East Fork Lake (red circle); Delaware Lake (green triangle); and Madison Lake (blue diamond).

A clustering dendrogram showed that the bacterial communities were clustered into four groups as follows: (1) a mixed cluster included one East Fork Lake sample (July 26), two Delaware Lake samples (June 11 and 16), and two Madison Lake samples (June 24 and July 6); (2) a cluster with seven East Fork Lake samples (June 21 and August 1, 2, 8, 15, 16, and 22); (3) a mixed cluster with three Delaware Lake samples (July 13, 20, and 21) and four Madison Lake samples (June 30, July 14, July 21, and July 27); and (4) a cluster with one Madison Lake sample on July 13 (Figure 5A). In the PCoA plot, the range of PC1 mostly differed for the water samples of East Fork Lake (0.0–0.4) compared with those of Delaware Lake and Madison Lake (−0.4–0.0). The PC2-values were rather dispersed, regardless of lake (Figure 5B). Results from both the PCoA plot and the clustering dendrogram showed that the spatial difference in bacterial community structure was more apparent in East Fork Lake compared to the other lakes. This suggests that both the variety and abundances of bacterial in East Fork Lake differed from those in the other two lakes, as the weighted PCoA plot utilizes a qualitative β diversity reflecting changes in how many sequences from each lineage are present, as well as changes in which taxa are present (Lozupone and Knight, 2005).

**Figure 6:**
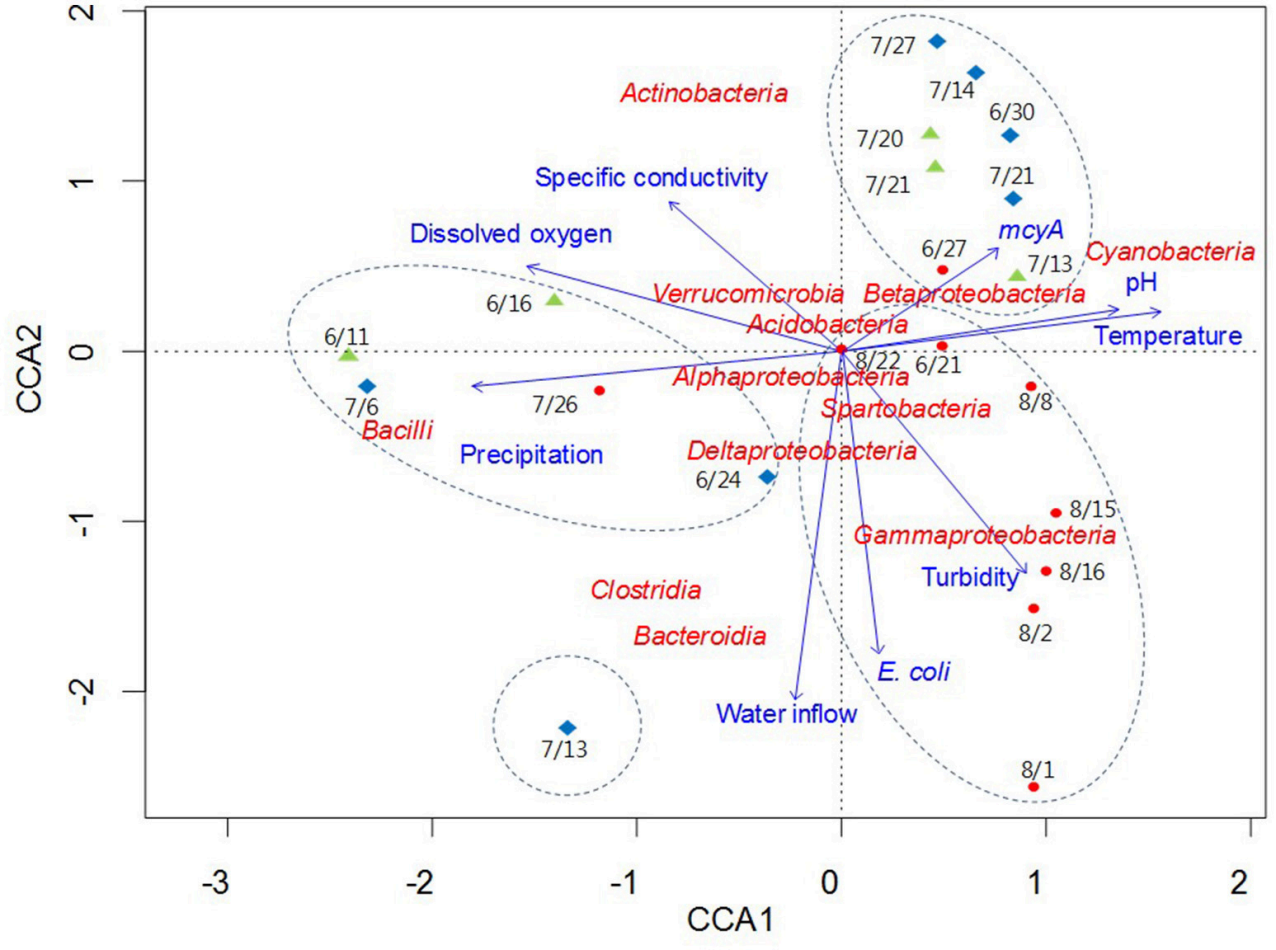
**Canonical correlation analysis of pyrosequencing data showing the relationships between bacterial communities and environmental parameters in three recreational freshwaters**. Arrows indicate the direction and magnitude of parameters associated with bacterial community structure. The length of each arrow in the ordination plot indicates the strength of the potential link between the environmental parameters and the composition of a certain bacterial community. Water samples from each lake are indicated with an appropriate sampling date and following symbols: East Fork Lake (red circle); Delaware Lake (green triangle); and Madison Lake (blue diamond). Dotted circles in the box indicate the clustering results by the UPGMA clustering dendrogram.

CCA was used to examine potential relationships between bacterial community composition and the environmental and microbial parameters measured in this study (Figure 6). Among the parameters, temperature, pH, dissolved oxygen, precipitation, and the *mcyA* genetic marker were the major components of the first axis in the ordination plot, explaining 71.3% of total variance, whereas water inflow, turbidity, and *E. coli* were major components of the second axis, explaining 21.1% of total variance. *Bacilli* and *Cyanobacteria* were separated from each other on the horizontal axis, while *Actinobacteria, Bacteroidia, Clostridia*, and *Gammaproteobacteria* were scattered on the vertical axis compared to other bacterial taxa located near the origin point (Figure 6). CCA showed strong correlations of *Clostridia* and *Bacteroidia* with water inflow on July 13 at Madison Lake, while *Gammaproteobacteria* were associated with turbidity on August 1 at East Fork Lake. In addition, the two bacterial classes, *Bacteroidia* and *Clostridia*, showed similar directions with that of *E. coli* in the CCA plot. On June 11 at Delaware Lake and July 6 at Madison Lake, *Bacilli* appeared to be linked with precipitation, while *Cyanobacteria* showed a positive association with temperature, pH, and the *mcyA* genetic marker.

## Discussion

### Bacterial community structure

The taxonomical assignment of the pyrosequencing dataset showed that the phyla of *Actinobacteria, Cyanobacteria, Firmicutes*, and *Proteobacteria* prevailed in the three lakes. *Actinobacteria* were previously regarded as typical inhabitants of soil environments. However, *Actinobacteria* have been recently reported as autochthonous bacteria in freshwater habitats (Warnecke et al., 2004), that can degrade organic polymers such as a lignin (Ghai et al., 2014), or uptake amino acids by metabolizing N-containing aromatic polymers such as a chitin (Salcher et al., 2010; Eckert et al., 2012). The most abundant member in the phylum *Actinobacteria* was the genus *Mycobacterium*, which has been reported to inhabit various aquatic environments by utilizing various hydrocarbons (Vaerewijck et al., 2005). *Actinobacteria* was also reported to contain complete sets of genes to uptake phosphorus by coding enzymes for the conversion reaction from inositol phosphates into acetyl-CoA (Ghai et al., 2014). The co-abundance of *Actinobacteria* and *Cyanobacteria* is often observed under the eutrophic conditions with high phosphorus in water. Recently, two novel lineages in *Actinobacteria*, acTH1 and acTH2, were detected as dominant bacteria from both the water and sediment of shallow cyanobacteria-blooming freshwater in China, Lake Taihu (Wu Q. L. et al., 2007; Ghai et al., 2014), where all sequences belonged to the acTH2 lineage were identified as the genus *Mycobacterium*.

At the genus level, *Synechococcus* and *Cyanobium* were dominant components of *Cyanobacteria* in East Fork Lake and Delaware Lake, while the genus *Planktothrix* prevailed in Madison Lake. Considering that East Fork Lake and Delaware Lake are more oligotrophic than Madison Lake (Marion et al., 2010), this contrasting result is not surprising. The nutrient status of a lake can be a critical factor in the community structure shift of *Cyanobacteria*. The high occurrences of *Synechococcus* and *Cyanobium* in East Fork Lake and Delaware Lake were consistent with those in other oligotrophic freshwater lakes (Postius and Ernst, 1999; Callieri and Stockner, 2002). A study of *Cyanobacteria* in Lake Erie reported that both *Synechococcus* and *Cyanobium* were not found *two* decades ago, but became predominant genera due to a shift from a eutrophic to oligo-mesotrophic status (Ouellette et al., 2006). In contrast, *Planktothrix* has been reported as a bacterial indicator of eutrophic or hypertrophic lakes (Lira et al., 2011).

The class *Bacilli* exhibited large fluctuations in the phylum *Firmicutes* among the three lakes, raising the question of a potential link between its ecological niche and selective environmental conditions. For example, the genus *Exiguobacterium* was the most dominant group in the class *Bacilli* and was more frequently detected in Delaware Lake and Madison Lake than in East Fork Lake (Figure 4). It was reported that *Bacilli* were the most active bacteria in synthesizing *de novo* DNA for the spore germination during the early stages of re-wetting events in dried river sediments. This suggests that *Bacilli* could become primary microbial colonizers, and then passively release planktonic bacteria into the freshwater in their vegetative form (Fazi et al., 2008). In addition, a comparative genomic study reported that *Exiguobacterium* could exist in wide temperature ranges as two distinct forms, psychrophilic and thermophilic (Vishnivetskaya et al., 2009). Delaware Lake has a wide inundation area due to its large watershed, with water level changes of up to 6 m, while Madison Lake is shallow with a maximum depth of <2 m. On the other hand, the second deepest lake in Ohio, East Fork Lake, has a steep and rocky shoreline, falling off rapidly into deep water with a maximum depth of 34 m (Ohio Department of Natural Resources, http://ohiodnr.gov/). Considering these ecological and environmental conditions, the *Exiguobacterium* are speculated to survive and grow in the wide temperature and moisture ranges in the watershed of Delaware Lake and the sediments of Madison Lake.

In addition, *Betaproteobacteria* are known as some of the most abundant bacteria among the phylum *Proteobacteria* in typical freshwater systems (Van der Gucht et al., 2005). However, *Betaproteobacteria* have been reported to decrease during a cyanobacterial bloom events (Bagatini et al., 2014). The changes of pH affected by cyanobacterial growth could have an influence on the bacterial community structure of *Betaproteobacteria*. Among all genera in the class *Betaproteobacteria, Polynucleobacter* were detected in nearly all of the water samples (95%, 20/21) and *P. acidiphobus* was the most dominant species in the genus *Polynucleobacter* (Supplementary Table 1). Previous studies reported that *P. acidiphobus* is abundant at high pH (Wu X. et al., 2007), and not found in acidic freshwaters (Hahn et al., 2011). Thus, the distinct occurrence of *P. acidiphobus* suggested that the differentiation of the *Betaproteobacteria* community was likely influenced by high pH conditions (about 9) due to the growth of cyanobacteria.

### Bacterial responses to environments

In East Fork Lake, temperature was an important environmental factor influencing the bacterial community structure, especially for *Synechococcus*, which was the most representative genus of *Cyanobacteria* GpIIa. This is in accordance with another study reporting that temperature is the most determinative environmental factor for the population of *Synechococcus* (Lindström et al., 2005).

The negative correlation between *Cyanobacteria* and *Bacilli* was attributed to relationships between their dominant component genera. The genus *Methylocystis* is a group of Gram-negative aerobic type II methanotrophic bacteria that utilize methane as their sole source of carbon and energy, which favors environments such as lake sediments containing relatively high levels of methane and low levels of dissolved oxygen (Auman et al., 2000). The genus *Arthrobacter* is regarded as a group of zymogenous bacteria inhabiting soil and rhizosphere, with fluctuating abundance in response to changing soil conditions (Cacciari and Lippi, 1987). *Exiguobacterium* has wide distributions in soil and aquatic environments, such as permafrost/contemporary surface soil (Vishnivetskaya and Kathariou, 2005), and freshwater/seawater (Rodrigues et al., 2009). *Planomicrobium* is often isolated from halophilic environments such as fermented seafood, marine mud, and coastal sediment (Jung et al., 2009). The reasons for the negative correlations between *Cyanobacteria* and *Bacilli* remain unknown. However, these four psychrophilic genera of *Bacilli* seem to prefer cold temperature conditions (Wagner, 2008), while *Cyanobacteria* seem to proliferate exponentially under higher temperature conditions. In this regard, a stratified sampling with shorter intervals is needed to verify the connections between *Cyanobacteria* and *Bacilli*.

### Potential public health concerns

Even though harmful algal blooms were not observed in the three lakes during the study period, *Cyanobacteria* GI were frequently found in East Fork and Madison lakes from the pyrosequencing analysis. Most of the *Cyanobacteria* GI sequences were assigned to the genus *Anabaena*. Interestingly, the *mcyA* genetic marker shared the same direction with the *Cyanobacteria* GI cluster at the genus level in the CCA plot (data not shown). *Cyanobacteria* IV and XI were rarely found in water samples from East Fork Lake, which were assigned to *Leptolyngbya* sp. and *M. aeruginosa*. In Madison Lake, *Planktothrix* was most abundant in the cyanobacterial community, which is one of potential toxin-producing cyanobacteria (Christiansen et al., 2003).

The clustering result of the pyrosequencing dataset was interesting in Madison Lake. The family *Enterobacteriaceae* was significantly higher in Madison Lake (8.9%) than in the other two lakes (0.4–1.5%). In the *Enterobacteriaceae, Escherichia* had the greatest occurrence on July 13 in Madison Lake. The genera *Bacteroides* and *Enterococcus* were also found on the same day, where ~230 sequences (4.1% of the total bacteria) were assigned to the genus *Enterococcus*. Although the genera *Bacteroides* and *Enterococcus* can be derived from environmental sources, they were identified as *B. fragilis* and *E. faecalis*, which can be frequently observed in animal and human fecal flora (Simpson et al., 2002). Even so, the precise time of a fecal contamination event is unclear due to the possibility of persistent DNAs from antecedent contaminations. However, it is likely that the bacterial community structure in Madison Lake on July 13 was affected by external bacterial loading via water inflows containing fecal contamination (Figure 6).

The relative abundance of particular taxonomic groups changed significantly between water samples. Some genera of bacteria showed significantly higher abundance in the three lakes during the period of this study (e.g., *Cyanobacteria, Mycobacterium, Methylocystis*, and *Legionella*), calling for further investigation regarding whether they can be listed as candidates for opportunistic and emerging pathogens threatening public health. There were numerous unidentified OTUs in the phyla *Actinobacteria* and *Proteobacteria*. Despite no evidence for the presence of pathogenic *Mycobacterium* spp. (e.g., *M. tuberculosis*), many OTUs assigned to *Mycobacterium* spp. were dominant components in the water samples, including *M. moriokaense, M. wolinskyi, M. brisbanense, M. arupense*, and *M. senegalense*. Recently, these environmental or non-tuberculous mycobacteria have been known to cause opportunistic infections in humans (Primm et al., 2004).

Moreover, more than 10% of all of the *Proteobacteria* sequences were assigned to unclassified *Legionellaceae* in this study, implying a certain presence of unknown *Legionellaceae* in the recreational freshwaters. Many *Legionella* species can exist in aquatic environments and they have become important agent relevant to public health (Fields, 1996). For example, more than 18 species of *Legionella* were reported to be related to patients with pneumonia (Bangsborg, 1997). To clarify the potential health risks in recreational freshwaters, further isolation and characterization of unidentified *Mycobacterium* and *Legionella* are warranted.

## Author contributions

JL conceived the research. CSL and MK analyzed the pyrosequencing data and carried out statistical analyses. CL conducted the qPCR experiment. ZY advised the manuscript. All authors have contributed to the manuscript and approved it.

## Funding

This work was supported by the Public Health Preparedness for Infectious Diseases program (http://phpid.osu.edu) and The Ohio State University Targeted Investment in Excellence program sponsored by the Offices of Academic Affairs, the President, and Research.

### Conflict of interest statement

The authors declare that the research was conducted in the absence of any commercial or financial relationships that could be construed as a potential conflict of interest.
