# Supplementary material for: The Microbiota of Recreational Freshwaters and the Implications for Environmental and Public Health
Source: Front Microbiol. 2016 Nov 17;7:1826. doi: 10.3389/fmicb.2016.01826 (PMC5112438; doi:10.3389/fmicb.2016.01826)
Supplement: Supplementary file 1 [file DataSheet1.DOCX]

Supplementary Material

**Recreational Freshwater Microbiome: Environmental and Public Health Implications**

**Chang Soo Lee,^¶^, Minseok Kim,^¶^, Cheonghoon Lee, Zhongtang Yu, Jiyoung Lee^*^**

*** Correspondence:** Jiyong Lee: lee.3598@osu.edu

# Supplementary Figures and Tables

## Supplementary Figures

**(A)** *E. coli*


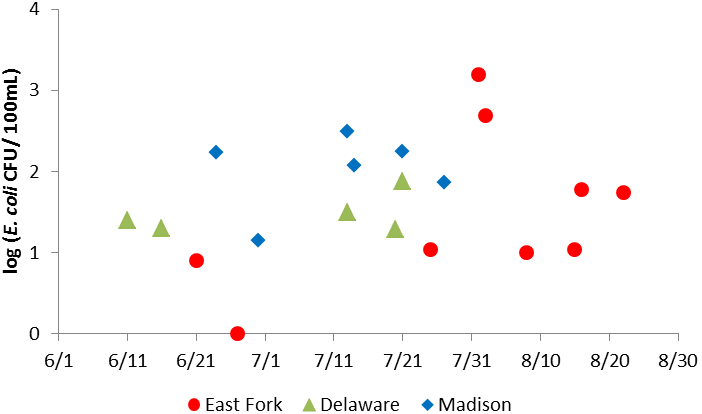


**(B)** *mcyA* genetic marker

**Supplementary Figure 1. Microbial water qualities in three lakes.** **(A)** *E. coli* and **(B)** *mcyA* genetic marker. Dotted line indicates the single-sample advisory limit of *E. coli* (235 CFU/ 100 mL) in freshwater beaches defined by the US Environmental Protection Agency advisory.

**Supplementary Figure 2. Standard curve of *mcyA* genetic marker.** DNA templates in the ranges of 4.4 × 10^0^-4.4 × 10^8^ gene copies/reaction were prepared from 10-fold serial dilutions of *M. aeruginosa*.

**Supplementary Table 1. The 20 most abundant representative OTUs of three recreational freshwaters.**

| Lake | representative OTU ID  (pyro tag ID) | % of sequence reads at each lake | Result of Identify Analysis | | | | | | |  |
| --- | --- | --- | --- | --- | --- | --- | --- | --- | --- | --- |
|  |  |  | Species | Strain | Accession | Sequence Similarity | Different/Total base | megaBLAST score | BLASTN score | E value* |
| East Fork | #276 (GPHQZAN04JDE3H) | 21.3% | *Synechococcus rubescens* | SAG 3.81 | AF317076 | 98.715 | 5/389 | 731 | 731 | 0.0  0.0 |
|  | #237 (GPHQZAN04IAVUP) | 10.6% | *Mycobacterium moriokaense* | DSM 44221(T) | AJ429044 | 99.688 | 1/320 | 626 | 626 | 1e-171  1e-167 |
|  | #3900 (GPHQZAN04I8UM3) | 6.0% | *Exiguobacterium acetylicum* | NCIMB 9889(T) | X70313 | 99.310 | 3/435 | 838 | 839 | 0.0  0.0 |
|  | #423 (GPHQZAN04ICACY) | 4.7% | *Streptoalloteichus tenebrarius* | NBRC 16177(T) | AB184722 | 92.429 | 24/317 | 430 | 0 | 6e-130  8e-133 |
|  | #2288 (GPHQZAN04ID8JJ) | 2.5% | *Acinetobacter johnsonii* | DSM 6963(T) | X81663 | 99.532 | 2/427 | 809 | 789 | 0.0  0.0 |
|  | #1502 (GPHQZAN04H76XI) | 2.2% | *Mycobacterium brisbanense* | ATCC 49938(T) | AY012577 | 98.113 | 6/318 | 583 | 583 | 4e-162  6e-160 |
|  | #4340 (GPHQZAN04JB3QQ) | 1.7% | *Paenisporosarcina macmurdoensis* | CMS 21w(T) | AJ514408 | 99.771 | 1/437 | 858 | 858 | 0.0  0.0 |
|  | #1182 (GPHQZAN04JA9IW) | 1.7% | *Pelagibacter ubique* | HTCC1062 | CP000084 | 88.630 | 44/387 | 422 | 404 | 1e-137  5e-143 |
|  | #4161 (GPHQZAN04IDOOX) | 1.2% | *Mycobacterium wolinskyi* | ATCC 700010(T) | Y12873 | 98.113 | 6/318 | 583 | 583 | 3e-162  6e-160 |
|  | #1782 (GPHQZAN04IP2R8) | 1.2% | *Mycobacterium senegalense* | CIP 104941(T) | AY457081 | 97.444 | 8/313 | 553 | 539 | 6e-155  8e-152 |
|  | #2441 (GPHQZAN04JW31X) | 1.0% | *Synechococcus rubescens* | SAG 3.81 | AF317076 | 96.907 | 12/388 | 646 | 605 | 0.0  6e-180 |
|  | #2641 (GPHQZAN04JU3MA) | 1.0% | *Chthoniobacter flavus* | Ellin428(T) | ABVL01000001 | 89.908 | 44/436 | 533 | 509 | 1e-167  3e-172 |
|  | #1918 (GPHQZAN04JI1HK) | 0.9% | *Schlegelella thermodepolymerans* | K14(T) | AY152824 | 97.152 | 9/316 | 555 | 555 | 5e-156  2e-154 |
|  | #4281 (GPHQZAN04IV0NT) | 0.8% | *Exiguobacterium undae* | DSM 14481(T) | DQ019165 | 99.770 | 1/435 | 854 | 854 | 0.0  0.0 |
|  | #464 (GPHQZAN04IVNG4) | 0.8% | *Polynucleobacter acidiphobus* | MWH-PoolGreenA3(T) | FM208180 | 99.682 | 1/314 | 614 | 615 | 3e-168  3e-164 |
|  | #1073 (GPHQZAN04IR5VV) | 0.7% | *Synechococcus rubescens* | SAG 3.81 | AF317076 | 95.833 | 10/240 | 390 | 369 | 6e-111  3e-108 |
|  | #3191 (GPHQZAN04JI7UA) | 0.7% | *Planktophila limnetica* | MWH-EgelM2-3.acI | FJ428831 | 89.801 | 41/402 | 462 | 448 | 4e-148  2e-154 |
|  | #336 (GPHQZAN04JCE09) | 0.7% | *Merismopedia tenuissima* | 0BB46S01 | AJ639891 | 98.294 | 5/293 | 505 | 472 | 9e-143  3e-138 |
|  | #1023 (GPHQZAN04JRFNZ) | 0.7% | *Mycobacterium arupense* | AR30097(T) | DQ157760 | 98.684 | 4/304 | 563 | 557 | 4e-156  7e-153 |
|  | #1491 (GPHQZAN04I2KE2) | 0.6% | *Fodinicola feengrottensis* | HKI 0501(T) | EF490376 | 92.429 | 24/317 | 454 | 438 | 6e-135  5e-135 |
| Delaware | #4340 (GPHQZAN04JB3QQ) | 21.1% | *Paenisporosarcina macmurdoensis* | CMS 21w(T) | AJ514408 | 99.771 | 1/437 | 858 | 858 | 0.0  0.0 |
|  | #237 (GPHQZAN04IAVUP) | 10.2% | *Mycobacterium moriokaense* | DSM 44221(T) | AJ429044 | 99.688 | 1/320 | 626 | 626 | 1e-171  1e-167 |
|  | #4161 (GPHQZAN04IDOOX) | 8.2% | *Mycobacterium wolinskyi* | ATCC 700010(T) | Y12873 | 98.113 | 6/318 | 583 | 583 | 2e-159  3e-157 |
|  | #1502 (GPHQZAN04H76XI) | 6.5% | *Mycobacterium brisbanense* | ATCC 49938(T) | AY012577 | 98.113 | 6/318 | 583 | 583 | 4e-162  6e-160 |
|  | #276  (GPHQZAN04JDE3H) | 6.3% | *Synechococcus rubescens* | SAG 3.81 | AF317076 | 98.715 | 5/389 | 731 | 731 | 0.0  0.0 |
|  | #4281 (GPHQZAN04IV0NT) | 4.8% | *Exiguobacterium undae* | DSM 14481(T) | DQ019165 | 99.770 | 1/435 | 854 | 854 | 0.0  0.0 |
|  | #2064 (GPHQZAN04IOUE8) | 3.2% | *Arthrobacter oryzae* | KV-651(T) | AB279889 | 99.344 | 2/305 | 581 | 575 | 6e-160  4e-156 |
|  | #3198 (GPHQZAN04H4IZC) | 2.8% | *Planomicrobium koreense* | JG07(T) | AF144750 | 99.083 | 4/436 | 833 | 833 | 0.0  0.0 |
|  | #423 (GPHQZAN04ICACY) | 1.7% | *Streptoalloteichus tenebrarius* | NBRC 16177(T) | AB184722 | 92.429 | 24/317 | 430 | 0 | 0.0  0.0 |
|  | #2090 (GPHQZAN04JHERA) | 1.6% | *Paenisporosarcina quisquiliarum* | SK 55(T) | DQ333897 | 98.169 | 8/437 | 795 | 789 | 6e-130  8e-133 |
|  | #1182 (GPHQZAN04JA9IW) | 1.0% | *Pelagibacter ubique* | HTCC1062 | CP000084 | 88.630 | 44/387 | 422 | 404 | 1e-137  5e-143 |
|  | #1023 (GPHQZAN04JRFNZ) | 1.0% | *Mycobacterium arupense* | AR30097(T) | DQ157760 | 98.684 | 4/304 | 563 | 557 | 4e-156  7e-153 |
|  | #4145 (GPHQZAN04IGB5U) | 1.0% | *Exiguobacterium artemiae* | 9AN(T) | AM072763 | 97.471 | 11/435 | 783 | 775 | 0.0  0.0 |
|  | #1195 (GPHQZAN04IP2E0) | 0.9% | *Paenisporosarcina macmurdoensis* | CMS 21w(T) | AJ514408 | 96.339 | 16/437 | 747 | 739 | 0.0  0.0 |
|  | #249 (GPHQZAN04JDP3C) | 0.8% | *Exiguobacterium mexicanum* | 8N(T) | AM072764 | 100.000 | 0/324 | 642 | 642 | 2e-175  2e-171 |
|  | #3229 (GPHQZAN04JBAX5) | 0.8% | *Fodinicola feengrottensis* | HKI 0501(T) | EF490376 | 94.006 | 19/317 | 478 | 478 | 6e-140  2e-141 |
|  | #2965 (GPHQZAN04I0H18) | 0.7% | *Paenisporosarcina macmurdoensis* | CMS 21w(T) | AJ514408 | 96.894 | 10/322 | 569 | 569 | 8e-159  3e-157 |
|  | #1305 (GPHQZAN04IPRIA) | 0.6% | *Paenisporosarcina macmurdoensis* | CMS 21w(T) | AJ514408 | 96.897 | 13/419 | 757 | 735 | 0.0  0.0 |
|  | #3420 (GPHQZAN04JVI9M) | 0.6% | *Methylocystis heyeri* | H2(T) | AM283543 | 97.165 | 11/388 | 674 | 668 | 0.0  0.0 |
|  | #2690 (GPHQZAN04ICIIS) | 0.6% | *Paenisporosarcina macmurdoensis* | CMS 21w(T) | AJ514408 | 97.465 | 11/434 | 731 | 690 | 0.0  0.0 |
| Madison | #1041 (GPHQZAN04H70ZI) | 11.2% | *Planktothrix rubescens* | BC-Pla 9401 | AJ132250 | 99.744 | 1/390 | 765 | 765 | 0.0  0.0 |
|  | #4281 (GPHQZAN04IV0NT) | 10.1% | *Exiguobacterium undae* | DSM 14481(T) | DQ019165 | 99.770 | 1/435 | 854 | 854 | 0.0  0.0 |
|  | #237 (GPHQZAN04IAVUP) | 8.8% | *Mycobacterium moriokaense* | DSM 44221(T) | AJ429044 | 99.688 | 1/320 | 626 | 626 | 1e-171  1e-167 |
|  | #423 (GPHQZAN04ICACY) | 6.5% | *Streptoalloteichus tenebrarius* | NBRC 16177(T) | AB184722 | 92.429 | 24/317 | 430 | 0 | 6e-130  8e-133 |
|  | #555 (GPHQZAN04IQFLA) | 5.9% | *Staphylococcus aureus subsp. aureus* | Mu50 | BA000017 | 99.771 | 1/436 | 856 | 856 | 0.0  0.0 |
|  | #4161 (GPHQZAN04IDOOX) | 5.4% | *Mycobacterium wolinskyi* | ATCC 700010(T) | Y12873 | 98.113 | 6/318 | 583 | 583 | 3e-162  6e-160 |
|  | #3198 (GPHQZAN04H4IZC) | 3.5% | *Planomicrobium koreense* | JG07(T) | AF144750 | 99.083 | 4/436 | 833 | 833 | 0.0  0.0 |
|  | #3420 (GPHQZAN04JVI9M) | 2.4% | *Methylocystis heyeri* | H2(T) | AM283543 | 97.165 | 11/388 | 674 | 668 | 0.0  0.0 |
|  | #2815 (GPHQZAN04JS7SC) | 1.9% | *Pseudomonas aeruginosa* | LMG 1242(T) | Z76651 | 99.532 | 2/427 | 831 | 831 | 0.0  0.0 |
|  | #3229 (GPHQZAN04JBAX5) | 1.4% | *Fodinicola feengrottensis* | HKI 0501(T) | EF490376 | 94.006 | 19/317 | 478 | 478 | 6e-140  2e-141 |
|  | #1491 (GPHQZAN04I2KE2) | 1.3% | *Fodinicola feengrottensis* | HKI 0501(T) | EF490376 | 92.429 | 24/317 | 454 | 438 | 6e-135  5e-135 |
|  | #3900 (GPHQZAN04I8UM3) | 1.2% | *Exiguobacterium acetylicum* | NCIMB 9889(T) | X70313 | 99.310 | 3/435 | 838 | 839 | 0.0  0.0 |
|  | #4340 (GPHQZAN04JB3QQ) | 1.1% | *Paenisporosarcina macmurdoensis* | CMS 21w(T) | AJ514408 | 99.771 | 1/437 | 858 | 858 | 0.0  0.0 |
|  | #3245 (GPHQZAN04JNZBD) | 0.1% | *Cryobacterium luteum* | Hh15(T) | HQ845193 | 96.393 | 11/305 | 509 | 504 | 5e-145  3e-144 |
|  | #464 (GPHQZAN04IVNG4) | 0.1% | *Polynucleobacter acidiphobus* | MWH-PoolGreenA3(T) | FM208180 | 99.682 | 1/314 | 614 | 615 | 3e-168  3e-164 |
|  | #4448 (GPHQZAN04IMEXK) | 0.1% | *Limnohabitans australis* | MWH-BRAZ-DAM2D(T) | FM178226 | 96.956 | 13/427 | 743 | 743 | 0.0  0.0 |
|  | #1502 (GPHQZAN04H76XI) | 0.1% | *Mycobacterium brisbanense* | ATCC 49938(T) | AY012577 | 98.113 | 6/318 | 583 | 583 | 4e-162  6e-160 |
|  | #2064 (GPHQZAN04IOUE8) | 0.1% | *Arthrobacter oryzae* | KV-651(T) | AB279889 | 99.344 | 2/305 | 581 | 575 | 6e-160  4e-156 |
|  | #1182 (GPHQZAN04JA9IW) | 0.1% | *Pelagibacter ubique* | HTCC1062 | CP000084 | 88.630 | 44/387 | 422 | 404 | 1e-137  5e-143 |
|  | #173  (GPHQZAN04IB0RZ) | 0.1% | *Bacteroides fragilis* | NCTC 9343(T) | CR626927 | 99.268 | 3/410 | 767 | 747 | 0.0  0.0 |

* Each E value was calculated based on megaBLAST (above) or BLASTN (below), respectively.
